# Internal but not external noise frees working memory resources

**DOI:** 10.1371/journal.pcbi.1006488

**Published:** 2018-10-15

**Authors:** Ivan Tomić, Paul M. Bays

**Affiliations:** 1 Faculty of Humanities and Social Sciences, Department of Psychology, University of Zagreb, Zagreb, Croatia; 2 Department of Psychology, University of Cambridge, Cambridge, United Kingdom; Uppsala Universitet, SWEDEN

## Abstract

The precision with which visual information can be recalled from working memory declines as the number of items in memory increases. This finding has been explained in terms of the distribution of a limited representational resource between items. Here we investigated how the sensory strength of memoranda affects resource allocation. We manipulated signal strength of an orientation stimulus in two ways: we varied the internal (sensory) noise by adjusting stimulus contrast, and varied the external (stimulus) noise by altering the within-stimulus variability. Both manipulations had similar effects on the precision with which the orientation could be recalled, but differed in their impact on memory for other stimuli. These results indicate that increasing internal noise released resources that could be used to store other stimuli more precisely; increasing external noise had no such effect. We show that these observations can be captured by a simple neural model of working memory encoding, in which spiking activity takes on the role of the limited resource.

## Author summary

Investigations of visual short-term memory typically involve memorising clearly visible objects with elementary features, such as monochromatic disks or oriented bars. Results of such studies indicate that memory is allocated like a limited resource, i.e. shared out between objects. However, in daily life we are often confronted with visual features that are difficult to make out, like when an object is in shadow, or poorly-defined, like the color of a variegated leaf. Here we asked whether these kinds of features occupy as much memory resource as simple highly-visible objects. Our results demonstrate that reducing the sensory strength of a stimulus makes the quality of recall worse, but also takes up less resource so other objects can be remembered more precisely. Increasing the variability within a stimulus worsens recall, but has no effect on how other objects are remembered. These findings can be explained by considering how visual information is stored in populations of neurons: only the manipulation of sensory strength changes the amount of spiking activity dedicated to a stimulus.

## Introduction

The fidelity with which items are stored in visual working memory (VWM) depends on the total number of elements in a visual scene. As the number of items increases, their representations in memory become increasingly variable, leading to less precise recall of each item [1–3]. These findings are consistent with a resource model of VWM, which describes a limited representational medium that is allocated to objects within a visual scene [1, 4–11]. Consistent with the resource concept, rather than an even storage resolution for all remembered objects, it has been shown that resources can be flexibly distributed. Both external (e.g. visual salience) and internal (e.g. behavioral relevance) factors can lead to enhanced storage resolution of an object, but with a corresponding cost to the fidelity of other items held in memory [12–19]. Note that some authors argue there is a fixed upper limit on the number of stimuli that working memory resources can be allocated to (e.g. [20]), but this is not a topic of the present study.

A recently proposed neural resource model [21, 22] has provided a biological account of behaviorally observed VWM limitations that is consistent with neurophysiological findings [23–25]. In this neural model, items are encoded in a population of tuned neurons that fire stochastically according to a Poisson process. The total activity in the neural population is constant across changes in set size, as the result of a global normalization mechanism (see [11] for further discussion and e.g. [26, 27] for neurophysiological evidence of analogous mechanisms). Therefore, increasing the set size leads to a decrease in the activity of neurons encoding each item, providing a biological basis for limited resources. As the neural signal representing each object decreases, internal representations become dominated by random noise in spiking activity, leading to a decrease in memory precision. Neural noise, an inherent feature of neural processing [28], therefore determines the fidelity of internal representations and represents the limiting factor on VWM performance.

Since storing each additional element in VWM takes resources away from existing items, it is of importance to understand what determines an object’s share of resource. One candidate as a limiting factor is the strength of the sensory stimulation, which determines the extent to which noise in the visual system can corrupt the stimulus representation. The importance of the visual system’s internal noise has long been recognized (e.g., [29]) and many studies have aimed to characterize its effects on detection and discrimination performance [30–34]. In a recent study [35], using an analogue report task with a single item, internal noise was modulated by varying the contrast of the orientation stimulus to be reported. Results showed precision declined as contrast decreased. A population coding model successfully accounted for the detailed pattern of errors by decreasing the total activity in the neural population. One intuitive prediction arising from this model is that, in the case of multiple items, the neural resource (spiking activity) released when storing a low contrast item could be used to store other items.

Often, efficient visual processing is not only hampered by internal noise in the visual system, but also by stimulus uncertainty (i.e. external noise) which is intrinsic to natural circumstances. The complexity of our visual environment is likely to preclude an even distribution of resources, but it is less clear how exactly stimulus variance affects resource allocation. It has been demonstrated that increasing the external (stimulus) noise leads to poorer performance in perceptual tasks [36–39]. For example, judging the average feature value from an array becomes less accurate as uncertainty increases, i.e. as the variance of elements in the relevant feature dimension increases (e.g. [39]). A recent study [40] demonstrated that external noise had an opposing effect to internal noise on perceptual biases, consistent with a model of efficient coding [41].

The present study aimed to investigate resource distribution among memoranda with differing sensory strengths and variabilities. Using a cued recall task we looked for changes in recall precision while manipulating the stimulus in order to vary the influence of either internal (Experiment 1) or external (Experiment 2) noise. We found similar effects of changing internal or external noise on recall of the noisy stimuli themselves. However, the consequences for other stimuli in memory differed, indicating that only changes to internal noise released neural resources that could be used to store other stimuli more precisely.

## Results

### Experiment 1

**Fig 1:**
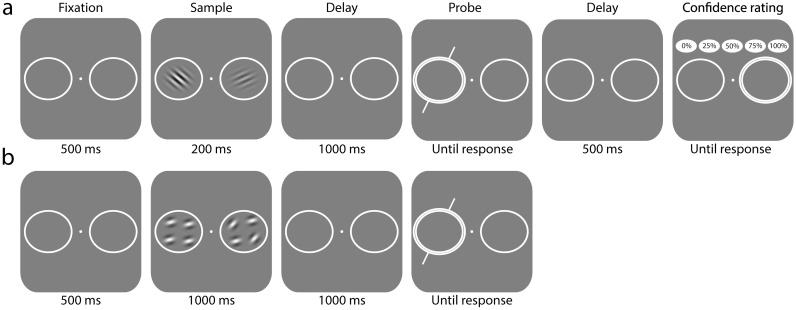
Experimental task. (a) Experiment 1. Two orientation stimuli were presented followed by a delay, then one location was probed at random and subjects adjusted the probe bar to match the remembered orientation at that location. Stimuli varied in contrast. Participants were subsequently asked for a confidence rating. (b) Experiment 2. Two composite stimuli were presented each containing four orientations. After a delay, one location was probed and participants reported the average orientation they recalled at that location. Stimuli differed in the variability of their component orientations.

**Fig 2:**
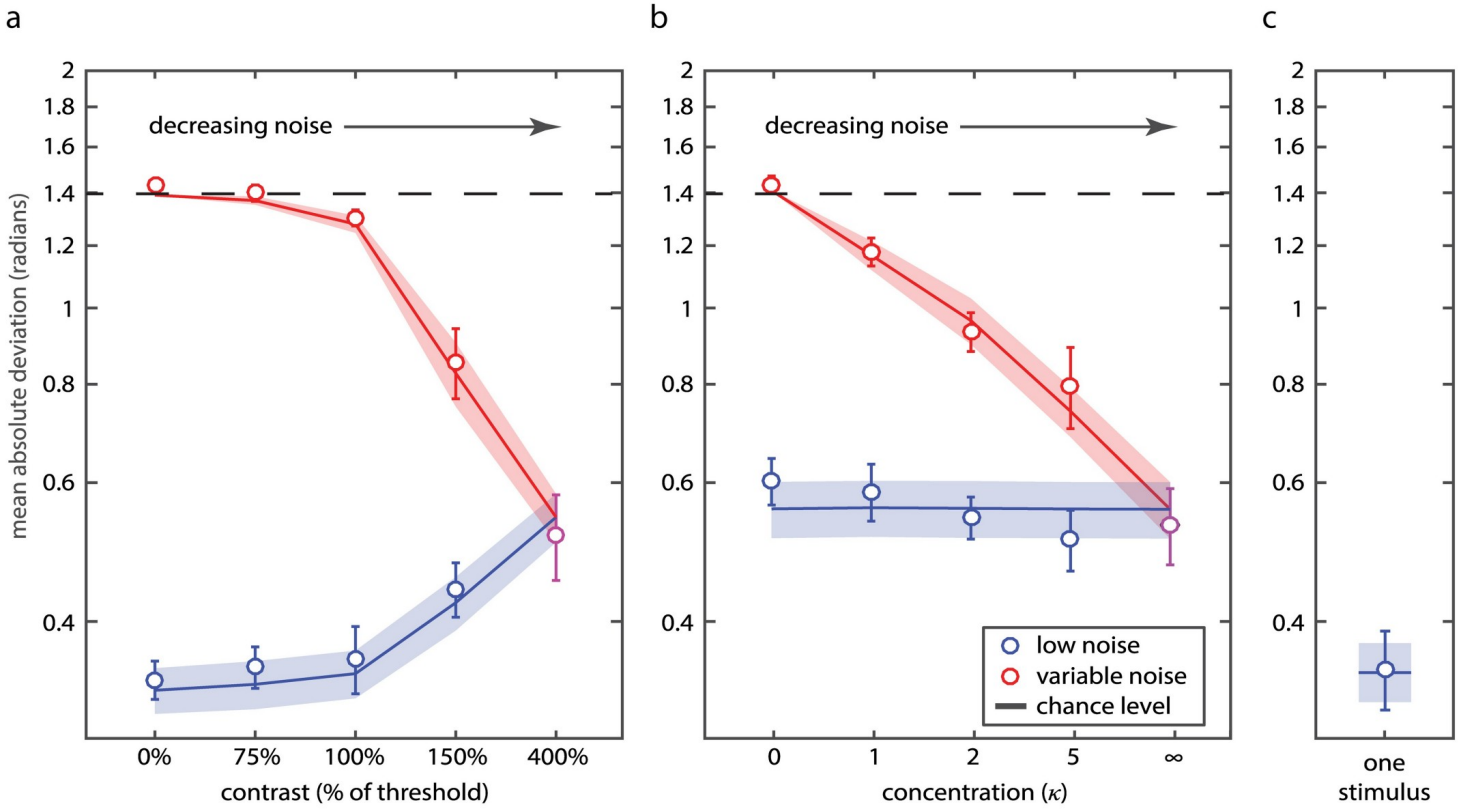
Recall variability. (a) Mean absolute deviation of responses as a function of contrast of the *variable noise* stimulus in Experiment 1. Empirical data for recall of *low noise* and *variable noise* stimuli are plotted as blue and red circles, respectively (error bars indicate ±1 SE). Blue and red curves show corresponding predictions of the neural resource model with ML parameters. (b) Recall variability as a function of concentration of the *variable noise* stimulus in Experiment 2. (c) Recall variability on single stimulus trials in Experiment 2. Dashed lines indicate chance level performance.

**Fig 3:**
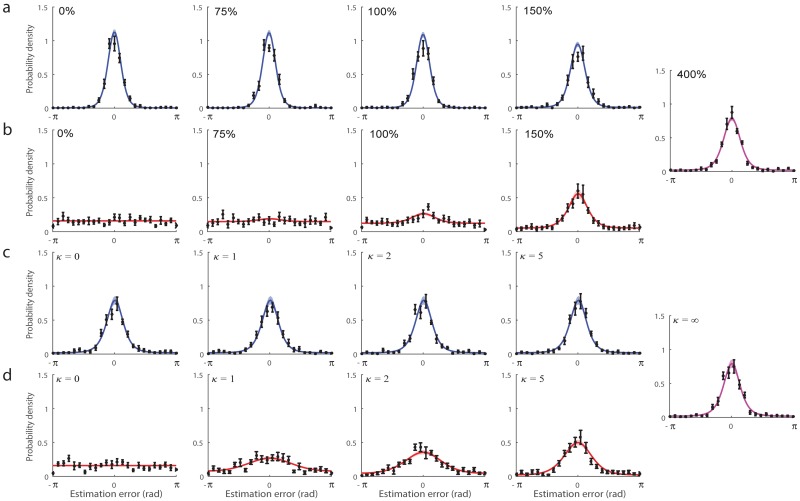
Recall errors and model fits. (a & b) Consequences of varying internal noise (Experiment 1): different panels correspond to different levels of contrast for the *variable noise* stimulus. Error distributions are plotted for the *low noise* stimulus (a, blue) and *variable noise* stimulus (b, red). Data points and errorbars show empirical recall errors (mean ±1 SE). Colored curves and patches show predictions of the neural resource model with ML parameters (mean ±1 SE). A single plot is shown far right for the condition in which both stimuli had the same (low) noise. (c & d) Consequences of varying external noise (Experiment 2): plots as in (a & b).

To investigate working memory resource allocation among stimuli affected to different degrees by internal noise, we presented participants with pairs of orientation stimuli of varying contrasts (Fig 1a). One stimulus on each trial had fixed high contrast while the contrast of the other varied as a percentage of each participant’s detection threshold. To capture performance on this task we calculated the mean absolute deviation of recall responses from the mean of the response distribution. Fig 2a depicts these values for *low noise* (blue circles) and *variable noise* (red circles) stimuli. Error distributions for each stimulus and contrast level are shown in Fig 3a & 3b. All distributions, except for the 0% and 75% contrast variable-noise stimuli, displayed significant central tendency (V > 63.8, p < 0.001).

Overall, participants performed better when recalling *low noise* than *variable noise* stimuli (F(1,8) = 839.7, p < 0.001, $\eta_{p}^{2}$ = 0.99; ANOVA, contrast × probed stimulus) and when total noise in a trial decreased (F(3, 24) = 14.3, p < 0.001, $\eta_{p}^{2}$ = 0.64). A significant stimulus contrast × probed stimulus interaction was observed (F(3, 24) = 37.4, p < 0.001, $\eta_{p}^{2}$ = 0.82) consistent with a benefit on *variable noise* stimulus recall and detriment on *low noise* stimulus recall as contrast of the *variable noise* stimulus increased.

To test this statistically, we compared recall performance on trials when both Gabors had high (400%) contrast with trials where contrast of one Gabor was zero. Recall of *variable noise* stimuli improved with increasing contrast (t(8) = 14.2; p < 0.001). Critically, recall performance for *low noise* stimuli worsened as the contrast of the *variable noise* stimulus increased (t(8) = 3.2, p = 0.013). These results indicate that increasing the internal noise associated with one stimulus has a beneficial effect on recall of the other stimulus, consistent with a transfer of working memory resources from the noisier to the less noisy stimulus.

#### Confidence ratings

Subjective confidence for the presence of the *variable noise* stimulus was significantly correlated with both stimulus contrast (r^2^ = 0.87, t(8) = 52.4, p < 0.001) and performance (r^2^ = 0.93, t(8) = 152, p < 0.001). Participants’ mean confidence ratings were: 0% contrast, mean rating 11%; 75%, 14%; 100%, 23%; 150%, 59%; 400%, 92%. Testing for association of performance and confidence ratings within each contrast condition showed significant correlations except for the lowest (stimulus present) and highest contrasts: 75% contrast, r^2^ = 0.06, t(8) = 1.53, p = 0.16; 100% contrast, r^2^ = 0.12, t(8) = 3.40, p = 0.009; 150% contrast, r^2^ = 0.15, t(8) = 8.14, p < 0.001; 400% contrast, r^2^ = 0.03, t(6) = 0.61, p = 0.56. In both 75% and 400% contrast conditions the absence of correlation can be explained by a strong restriction in the range of confidence ratings used, with 65.2% and 77.5% of trials classified as 0% and 100% confident in 75% and 400% contrast conditions, respectively.

#### Neural resource model fits

We fit recall errors with a neural resource model based on population coding (Fig 5) [21, 22, 35]. In the model, total activity of neurons encoding both stimuli is normalized, such that a fixed amount of spiking activity is shared between stimuli. Curves in Figs 2a and 3a & 3b show fits of the model with maximum likelihood (ML) parameters (population gain *γ* = 58.5 Hz ± 12.1 Hz, tuning width *κ* = 2.48 ± 0.26, response bias *β* = –0.004 rad ± 0.020 rad, contrast function parameters *σ* = 1.78 ± 0.17 and *α* = 9.09 ± 2.66). The model provided an excellent quantitative fit to both mean angular deviation (mean individual r^2^ = 0.94; range 0.89–0.97; aggregated data r^2^ = 0.97; Fig 2a) and response histograms (Fig 3a & 3b), successfully capturing the trade-off in recall performance between *low noise* and *variable noise* stimuli with changes in noise level.

#### Sequential presentation

**Fig 4:**
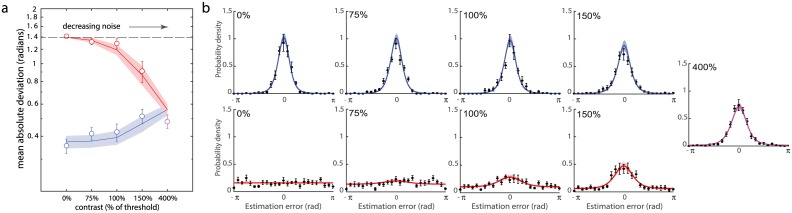
Sequential presentation experiment. (a) Mean absolute deviation of responses as a function of contrast of the *variable noise* stimulus. Empirical data for recall of *low noise* and *variable noise* stimuli are plotted as blue and red circles, respectively (error bars indicate ±1 SE). Blue and red curves show corresponding predictions of the neural resource model with ML parameters. Dashed line indicates chance level performance. (b) Recall errors and model fits. Consequences of varying internal noise: different panels correspond to different levels of contrast for the *variable noise* stimulus. Error distributions are plotted for the *low noise* stimulus (a, blue) and *variable noise* stimulus (b, red). Data points and errorbars show empirical recall errors (mean ±1 SE). Colored curves and patches show predictions of the neural resource model with ML parameters (mean ±1 SE). A single plot is shown far right for the condition in which both stimuli had the same (low) noise.

It is possible that, because the two simultaneously presented stimuli differed in salience, visual attention may have been automatically drawn to the higher contrast stimulus, resulting in a more detailed encoding. In other words, the observed effects could be due to competition for attentional rather than memory resources. To address this we conducted a follow-up experiment in which *low noise* and *variable noise* stimuli were presented sequentially (see Methods). The results confirmed the key findings of the original experiment in which items were presented simultaneously, providing evidence against competition for attention as the basis for our results. Fig 4a depicts mean absolute deviations for *low noise* (blue circles) and *variable noise* (red circles) stimuli, while Fig 4b shows error distributions for each stimulus and contrast level. All distributions, except for the 0% contrast variable-noise stimuli, displayed significant central tendency (V > 30.1, p < 0.019).

Specifically, participants showed better performance when recalling *low noise* than *variable noise* stimuli (F(1,9) = 168.4, p < 0.001, $\eta_{p}^{2}$ = 0.95; ANOVA, contrast × probed stimulus) and when total noise in a trial decreased (F(3, 27) = 4.8, p < 0.01, $\eta_{p}^{2}$ = 0.35). Again, the stimulus contrast × probed stimulus interaction was significant (F(3, 27) = 13.1, p < 0.001, $\eta_{p}^{2}$ = 0.59) reaffirming a benefit to *variable noise* stimulus recall and detriment to *low noise* stimulus recall as the *variable noise* stimulus strengthened.

The significant interaction was further explored by comparing performance between trials on which both Gabors had high (400%) contrast with trials where contrast of one Gabor was zero. As the contrast of the *variable noise* stimulus increased, recall of *variable noise* stimuli improved (t(9) = 17.8; p < 0.001) and recall of *low noise* stimuli worsened (t(9) = 3.8, p < 0.01), just as observed with simultaneous presentation.

Next, we tested for effects of presentation order on recall performance. A repeated measures ANOVA (contrast × presentation order) of *low noise* stimulus recall showed a significant effect of contrast level (F(4, 36) = 5.4, p < 0.01), but no significant effect of presentation order (F(1, 9) = 1.72, p = 0.22) nor any interaction (F(4, 36) = 1.26, p = 0.30). The same results were obtained when analyzing *variable noise* stimulus recall. ANOVA showed a significant effect of contrast level (F(3, 27) = 11.46, p < 0.001) but non-significant effects of presentation order (F(1, 9) = 0.52, p = 0.49) and no interaction (F(3, 27) = 2.29, p = 0.10).

#### Neural model

We fit the data with the same neural resource model used for the simultaneous-presentation task. Fits are shown as curves in Fig 4a & 4b (ML parameters: population gain *γ* = 102.95 Hz ± 17.54 Hz, tuning width *κ* = 1.54 ± 0.26, response bias *β* = 0.013 rad ± 0.014 rad, contrast function parameters *σ* = 1.96 ± 0.37 and *α* = 9.56 ± 2.41).

#### Encoding failures

The neural resource model followed [35] in assuming that the activity associated with a stimulus is a continuous (Naka-Rushton) function of contrast. In the previous study this model was shown to provide a superior account of recall data (for a single item) than a probabilistic encoding model, in which contrast affected the probability of encoding a stimulus in an all-or-nothing fashion. Although orthogonal to the purposes of the present study, we nonetheless examined this and several other related models based on the principle that the *variable noise* stimulus might fail to be encoded on some subset of trials (see Supporting Information for full methods and results). Formal model comparison favored the neural resource model over all alternatives tested, for both simultaneous and sequential versions of Exp 1.

### Experiment 2

The goal of this experiment was to explore working memory resource allocation among stimuli with differing amounts of external noise. On each trial we presented participants with two composite stimuli each consisting of a set of Gabors of differing variance. For one, *low noise* stimulus, within-set variance was always zero (all Gabors were parallel), while orientations for the other, *variable noise* stimulus were sampled from distributions of different widths. Participants were required to report from memory the average orientation of a probed stimulus.

Fig 2b plots performance for *variable noise* (red circles) and *low noise* (blue circles) stimuli. Error distributions for each stimulus and contrast level are shown in Fig 3c & 3d. All distributions, except for the zero precision (random) variable-noise stimulus, displayed significant central tendency indicating better-than-chance performance (V > 122.2, p < 0.001). Recall was more precise for *low noise* than *variable noise* stimuli (F(1,9) = 201.5, p < 0.001, $\eta_{p}^{2}$ = 0.96), and when the total amount of noise on a trial decreased (F(3,27) = 28.7, p < 0.001, $\eta_{p}^{2}$ = 0.76). A significant stimulus contrast × probed stimulus interaction was again observed (F(3,27) = 16.37, p < 0.001, $\eta_{p}^{2}$ = 0.65).

Next, we compared performance on trials with contrasting amounts of noise, i.e. *variable noise* stimuli in which all orientations were alike (*κ* = ∞) versus those drawn from a uniform distribution (*κ* = 0). A decrease in the noise level of the *variable noise* stimulus increased recall performance for that stimulus (t(9) = 13, p < 0.001), but had no effect on recall of the other, *low noise* stimulus (t(9) = 1.52, p = 0.16). In contrast to the findings of Exp 1, therefore, changing the amount of external noise for one stimulus did not influence recall of the other, consistent with a fixed resource allocation in the presence of changing stimulus noise.

We examined whether participants’ estimates were biased towards particular elements of composite stimuli. To this end, we distinguished the four elements in each set by location relative to fixation: horizontal near, horizontal far, vertical above, vertical below. We observed no significant differences in the mean absolute deviation of responses around the orientations of the different component elements (location × noise level ANOVA; location: F(3,27) = 0.8, p = 0.51; interaction: F(9,81) = 1.7, p = 0.10), indicating that participants were not systematically biased towards particular elements, and suggesting that participants sampled all the elements on a roughly equal basis to estimate the average orientation of the stimulus.

#### Single stimulus trials

Orientation recall on trials with a single *low-noise* stimulus was significantly better than recall of *low noise* stimuli in any of the two-stimulus conditions (Fig 2c; t(9) > 4.67; p < 0.001). We compared performance on single stimulus trials in Exp 2 with performance on trials in Exp 1 where contrast of the *variable noise* stimulus was zero. The difference did not reach significance (t(17) = 0.09; p = 0.92). These results indicate that the task of remembering a single composite stimulus in Exp 2 was comparable to the task of remembering a single high contrast orientation stimulus in Exp 1.

#### Comparison with Experiment 1

To confirm the pattern of results differed significantly between Exps 1 and 2, we performed an ANOVA on recall of *low noise* stimuli on trials with contrasting levels of *variable noise* stimulus (lowest and highest). We found a significant interaction between noise level and experiment (F(1,17) = 11.68, p < 0.01). This interaction was driven by a non-significant difference in performance between the two lowest noise conditions (i.e. 400% contrast and *κ* = ∞; t(17) = 0.08, p = 0.94) coupled with a significant difference between highest noise conditions (i.e. 0% contrast and *κ* = 0; t(17) = 5.52, p < 0.001).

#### Model fits

The neural resource model treats storing the average of a set of orientations identically to storing a single orientation stimulus. It assumes that the averaging process introduces variability in the stored value and this is proportional to the variability in the sample orientations. ML fits of the model are shown as curves in Figs 2b and 3c & 3d (population gain *γ* = 98.1 Hz ± 73.9 Hz, tuning width *κ* = 2.29 ± 0.32, response bias *β* = 0.014 rad ± 0.013 rad, scaling parameter *s* = 0.898 ± 0.135). The model again provided an excellent fit to data, capturing both mean angular deviation (mean individual r^2^ = 0.88, range 0.72–0.97; aggregated data r^2^ = 0.96; Fig 2b) and response histograms (Fig 3c & 3d), and successfully reproduced the effect of external variability on *variable noise* stimulus recall while displaying no effect on *low noise* stimuli.

## Discussion

Recent research has established that, as the working memory resource available to store an item decreases (e.g. as the number of items increases), recall of that item becomes more noisy. Here we address the converse question: does storing a stimulus with a greater level of noise require less resource? We considered two methods of manipulating the noise associated with a stimulus: (1) decreasing the sensory strength of the stimulus, which increases the influence of internal noise, and (2) increasing the intrinsic variability of a stimulus, a modulation of external noise.

We found that both noise manipulations had similar effects on the fidelity with which a stimulus could be recalled. However, they differed in their effects on recall of other items in memory. We found that increasing internal noise in one stimulus, by decreasing its contrast, had a favourable effect on recall of another (low-noise) stimulus. Increasing external noise, by raising internal variability of a stimulus, had no effect on memory for another stimulus. These results indicate that increases in internal, but not external, noise reduce the resource required to store a stimulus.

To account for these patterns of performance, we fit a variant of the neural resource model [21] to the behavioral data. In this model, stimulus features are encoded in spiking activity of a neural population. The total population activity is fixed, representing a finite resource that can be distributed between memoranda. This model has previously been shown to successfully reproduce the patterns of human recall error observed with changes in set size and shifts in stimulus priority [21].

When the contrast of a stimulus decreased, the firing rate associated with that stimulus in the model decreased as well, matching typical behavior of visual neurons in the brain. As a consequence, the representation was more influenced by stochasticity in spiking activity, and this had the consequence of poorer recall performance for that stimulus. Furthermore, due to normalization, the reduced activity devoted to the low contrast stimulus had the contingent effect of enhancing activity for another stimulus stored alongside it. In other words, dedicating less of the fixed neural activity to encoding the weak signal released resources that could be used to encode another signal more precisely. This model successfully captured not only the trade-off in precision between items of different sensory strengths (Fig 2), but also the detailed pattern of response errors in recall of each stimulus (Fig 3).

Next, we used the same population coding model to fit the observations under different levels of stimulus variability (external noise). Previous studies indicate that forming ensemble representations is a fundamental feature of the visual system [42–45], and that extracting summary statistics, such as averages, from a visual scene occurs automatically [46]. Here, we hypothesised that the averaging process would introduce variance in the stored estimate that was proportional to the variance in the samples, but have no effect on the overall firing rate of neurons encoding the stimulus. Storage of the calculated average orientation in working memory was treated identically to storing a single stimulus orientation. With these assumptions, the neural resource model successfully captured behavioral observations from the experiment. Increasing levels of external noise decreased the precision with which a stimulus could be recalled, but did not necessitate unequal allocation of neural activity, meaning that there was no contingent effect on other, simultaneously-stored stimuli. Note that at least one prediction of this account, that an averaged stimulus is represented with the same level of activity regardless of the variability in its component values, would be open to testing using single-cell or imaging techniques.

The two experiments differed slightly in the placement of stimuli in the visual field, in that the orientation stimuli in Exp 2 were presented on average a little more peripherally, and over a broader range of eccentricity, than the stimuli in Exp 1. Given that eccentricities of the two memory items presented on each trial were always equal, we are not aware of any reason why stimulus eccentricity would influence resource distribution between them, nor of any related mechanism that could account for the qualitative difference in results between Exps 1 and 2.

Previously it has been suggested that errors in working memory tasks can be explained by a doubly stochastic model, in which there is variability in the precision with which items are represented [5–7, 47]. Although the population coding model has only one source of stochasticity, a mathematical similarity between variable precision and the population coding model has been highlighted previously [21], and may explain the success of variable precision models in reproducing empirical error distributions. Because a process for generating variability is not specified in these models, however, they make no clear predictions about the effects of stimulus strength on performance: it is therefore difficult to see how they could account for the present results in anything but an *ad hoc* fashion.

At a more abstract level, it has been suggested that working memory can be viewed as a fixed capacity information channel [48]: this accounts for decreases in precision with set size and, with an appropriate choice of loss function, even predicts the specific error distributions observed in recall tasks [49] (although not currently swap errors; see below). Like the variable precision model, this model may be best seen as operating at a different conceptual level rather than being a competitor to the neural resource model. Unlike the neural resource model, this model cannot offer an explanation as to why less information is stored about a weaker stimulus, but it may be able to account for the consequence that more information is stored about other stimuli.

Swap errors, in which a wrong (i.e. unprobed) item is reported, are an important component of working memory performance at higher set sizes and when spatial confusability is high [4, 50, 51]. However, they are typically found to be vanishingly rare when, as here, only two well-separated items are presented for recall. A recent extension to the neural resource model based on conjunctive coding has demonstrated that swap errors can be explained using the same neural mechanism as response variability [22]. Future work using larger stimulus arrays could examine the effects of stimulus strength on swap errors.

We considered the possibility that the precision trade-off between low and high contrast stimuli in Exp 1 arose from biasing of visual attention towards the higher contrast item due to its greater perceptual salience. We repeated the experiment with a sequential mode of presentation, such that only one stimulus was visible on the screen at a time. As with simultaneous presentation, the results showed that recall precision for a stimulus of fixed high contrast varied with the contrast of the other stimulus presented on the same trial, confirming that our effects are due to competition for memory, rather than attentional, resources. This is consistent with previous results demonstrating that resources are distributed between memory stimuli similarly when presented sequentially as when presented simultaneously [14].

The seemingly continuous decline in recall precision of *low noise* stimuli with increasing *variable noise* contrast could reflect a mixture of trials in which (1) the *variable noise* stimulus was not encoded and the *low noise* stimulus was stored with high precision, and (2) both stimuli were encoded and precision of the *low noise* stimulus was thereby reduced, according to resource principles. Although this alternative account of the data would be no less consistent with allocation of a limited memory resource, we nonetheless addressed this possibility by fitting a number of alternative models that incorporated encoding failures in different ways (see Supporting Information). Consistent with results of [35], we found that the neural resource model had a consistent advantage in accounting for the data in each case.

Whether or not there exists a threshold for conscious perception is a debate with a lengthy history and a substantial existing literature, which we will not attempt to review here. Threshold models of various kinds have typically not fared well in comparison to signal detection theory in capturing psychophysical performance [52], but there is continuing disagreement in the field [53–55]. While the present results of model comparison, like those of [35], are consistent with a graded view of perception, in which even the weakest signals are (weakly) encoded, we do not imagine the present study can provide a definitive resolution to this question.

In summary, using a cued recall task and manipulating the signal strength of memoranda, we found that changing levels of internal, but not external noise, frees working memory resources, which can then be allocated to other items in the visual scene. These results are consistent with a neural resource model of working memory, in which representational fidelity is limited by a fixed total activation in neural populations encoding the stimuli.

## Methods

### Ethics statement

The study was approved by the Cambridge Psychology Research Ethics Committee.

### Participants

Thirty-one participants (10 males, 21 females; aged 18–33) took part in the study after giving informed consent in accordance with the Declaration of Helsinki. All participants reported normal color vision and normal or corrected-to-normal visual acuity.

### Apparatus

Stimuli were presented on a 21-inch gamma-corrected CRT monitor with a refresh rate of 60 Hz. The monitor was fitted with a neutral density filter to decrease the luminance range to the level of human detection thresholds. Participants viewed the monitor in a dark room at a distance of 50 cm, with head stabilized by a forehead and chin rest. Eye position was monitored online at 1000 Hz using an infrared eye tracker (SR Research). Stimulus presentation and response registration were controlled by a script written in Psychtoolbox 3.0.14 (Pelli, 1997) and run using Matlab 2016b (The Mathworks Inc.).

### Experiment 1

Ten participants (3 males, 7 females; aged 20–29) took part in Experiment 1. In this experiment, we tested how internal noise affects working memory performance by manipulating stimulus contrast.

Visual stimuli consisted of randomly oriented Gabor patches (wavelength of sinusoid, 0.75° of visual angle (1.33 cpd); s.d. of Gaussian envelope, 0.75°) presented on a grey background. A central fixation dot and two circles (white, 2° radius), located 5° to the left and to the right of the fixation dot were present throughout each trial. The circles served as placeholders for the Gabor patches.

We first obtained a detection threshold for each participant using the adaptive Psi method [56]. On each trial (100 in total) a Gabor patch of random orientation was presented for 200 ms within one of the two placeholder circles, randomly chosen; participants reported the side on which they saw the stimulus by a mouse click in the area inside the placeholder. Detection threshold was determined as the contrast level corresponding to 75% correct performance, estimated by fitting a cumulative normal function to the data. Contrast level on each trial was chosen to maximize the expected gain in information of the fitted psychometric parameters.

The main part of Experiment 1 was a cued recall task that tested memory for orientation (Fig 1a). Each trial started with presentation of the central fixation dot (gray) and placeholders. Once a stable fixation was recorded, the fixation dot turned white for 500 ms, followed by presentation of a memory display consisting of two randomly oriented Gabor patches for 200 ms. The contrast level of one patch (randomly chosen) was always 400% of the previously obtained detection threshold (*low noise* stimulus). The contrast level of the other Gabor was chosen at random from {0%, 75%, 100%, 150%, 400%} of detection threshold (*variable noise* stimulus).

After a 1000 ms blank retention interval, one of the two stimuli was randomly cued for recall. The cue consisted of a second, larger circle drawn around one of the placeholders and overlaid with a randomly-oriented bar. Using a mouse, participants adjusted the bar orientation to match the remembered orientation corresponding to the cued location. Once participants had made their response, a second cue was presented at the location of the *variable noise* patch and participants indicated how confident they were that a patch had been presented at that location by clicking on one of five buttons labelled {0%, 25%, 50%, 75%, 100%}.

Trials on which gaze deviated more than 2° from the fixation dot before the cue was presented were restarted with new random orientations. Participants completed 400 trials. One participant was excluded from analysis because data indicated poor comprehension of the confidence task instructions (uniform distribution of confidence ratings across all contrasts).

#### Sequential experiment

We performed a follow-up experiment based on Exp 1 but intended to minimise attentional competition between stimuli. Ten new participants (3 males, 7 females; aged 20–30 years) participated in the experiment. Experimental setup and procedure were identical to Exp 1 with the exception that the two memory stimuli on each trial were presented sequentially, for 200 ms each, separated by a 200 ms blank. For each participant the order of stimulus presentation was consistent throughout the experiment, either left-then-right or right-then-left, with counterbalancing across participants. This was done to remove any unpredictability as to where attention should be directed during a trial. To ensure temporal predictability, the first stimulus on each trial was presented at a fixed delay of 500 ms from the onset of the fixation dot. The order of *low noise* and *variable noise* stimuli was chosen at random on each trial. Eye movements were not recorded in this experiment.

### Experiment 2

Eleven participants (4 males, 7 females; aged 18–33 years) took part in Experiment 2. In this experiment we examined how external noise affected working memory performance by manipulating stimulus variability. Except where indicated, the procedure and stimulus timing were identical to Exp 1.

Stimuli consisted of randomly oriented Gabor patches (wavelength of sinusoid, 1.5° of visual angle (0.67 cpd); s.d. of Gaussian envelope, 0.9°) assembled in sets of four and presented on a grey background. Throughout the trial a central fixation dot and two placeholder circles (white, 3.5° radius), located 6° to the left and right of the fixation dot, were present.

On each trial, two sets of Gabor patches were presented for 1000 ms, one in each placeholder circle (Fig 1b). Participants were required to memorize the mean orientation of each set. The four patches within each set were positioned with equal spacing on an invisible circle (radius 2°), centered inside the placeholder and rotated randomly from trial to trial.

The variability of one set (left or right, randomly selected) was fixed at zero, i.e. all constituent Gabor patches had the same orientation (*low noise* stimulus). The variability of the other set (*variable noise* stimulus) was determined by sampling orientations from a von Mises distribution with randomly-assigned mean and concentration (inverse variability) chosen from {0, 1, 2, 5, ∞}. Note that a von Mises with concentration of zero is equivalent to a uniform distribution, i.e. patches had random orientations, while an infinite concentration meant that all patches had the same orientation.

Cue and response were the same as in Exp 1. Confidence ratings were not obtained in Exp 2. Participants completed 400 trials with two sets of Gabors as described above. On an additional 40 trials (randomly interleaved), to obtain a measure of baseline performance, a single *low noise* set was presented (randomly left or right) and cued for recall. One participant was excluded due to reporting on debriefing that they had ignored high noise stimuli.

### Analysis

Orientations were analysed and are reported with respect to the circular parameter space of possible values, i.e. the space of possible orientations [−90°, 90°) was mapped onto the circular space [−*π*, *π*) radians. Recall variability was assessed as the mean absolute deviation of recall errors from the mean of the error distribution. Tests of central tendency were performed using the V test [57] on pooled data within each condition. When testing model fits, estimates of the parameters were obtained separately for each subject over all experimental conditions using a nonlinear optimization algorithm (*fmincon* in MATLAB).

### Population coding model

**Fig 5:**
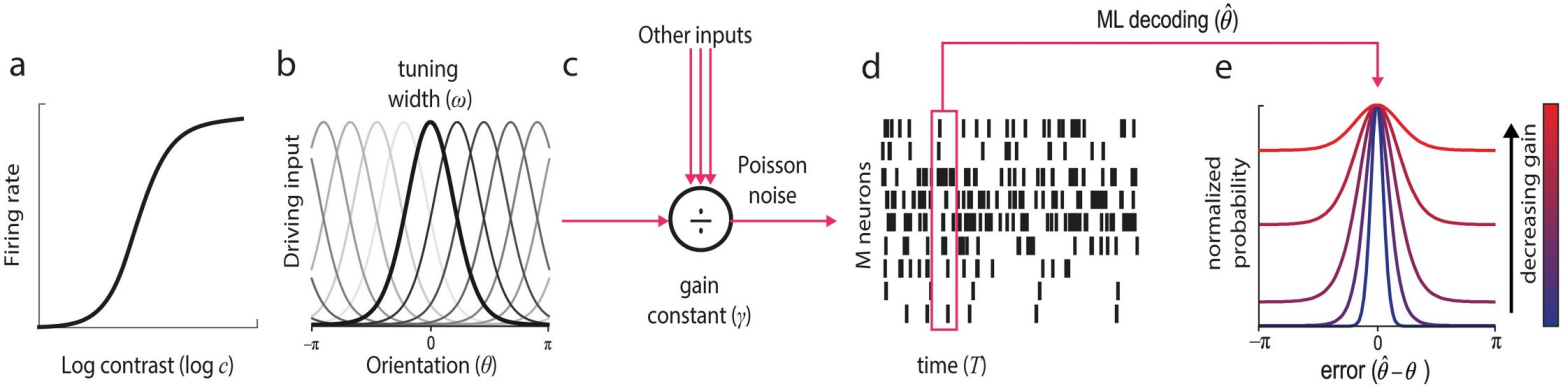
The neural resource model. (a & b) Stimulus features were encoded in the activity of idealized contrast sensitive and orientation selective neurons; preferred orientations were evenly distributed in orientation space. (c) Normalisation operated across the whole population, scaling summed activity to a fixed level determined by a gain constant. (d) Neurons generated spikes stochastically according to a Poisson process, with mean firing rate determined by the normalized input of each neuron. Orientation recall was modeled as maximum likelihood decoding of the spiking activity over a fixed time window. (e) Examples of error distributions predicted by the neural resource model.

We modeled orientation and contrast information presented at each stimulus location as providing input to an independent subpopulation of *M* neurons (Fig 5). The unnormalized response of the *i*th neuron responding to the *j*th stimulus was defined as the product of a von Mises (bell-shaped) orientation tuning function and a Naka-Rushton contrast response function:

$$\begin{array}{c} f_{ij}\left(\theta_{j},c_{j}\right)=g_{ij}\left(\theta_{j}\right)h_{j}\left(c_{j}\right), \end{array}$$ (1)

$$\begin{array}{c} g_{ij}\left(\theta_{j}\right)=\frac{1}{I_{0}\left(\kappa \right)}\exp\left(\kappa \cos\left(\theta_{j}-\varphi_{ij}\right)\right), \end{array}$$ (2)

$$\begin{array}{c} h_{j}\left(c_{j}\right)=\frac{c_{j}^{\alpha }}{\sigma^{\alpha }+c_{j}^{\alpha }}, \end{array}$$ (3)

where *θ*_*j*_ is the orientation of the *j*th stimulus, *c*_*j*_ is its contrast, *φ*_*ij*_ is the neuron’s preferred orientation, *κ* is a scale parameter for the tuning function, *σ* and *α* are parameters of the contrast response function, and *I*_0_(⋅) is the modified Bessel function of the first kind with order zero. Preferred orientations were evenly distributed throughout the circular space of possible values.

Normalization operated over the entire population of neurons, such that the post-normalization output of a neuron (its firing rate) was given by:

$$\begin{array}{c} r_{ij}=\frac{\gamma }{M}\frac{f_{ij}\left(\theta_{j},c_{j}\right)}{\sum_{k\ell }f_{k\ell }\left(\theta_{\ell },c_{\ell }\right)}, \end{array}$$ (4)

where *γ* is the population gain (i.e. summed population activity). Assuming that the distribution of tuning curves provides a dense uniform coverage of the orientation space (valid for large *M*), the summed activation of the population is independent of stimulus orientation and Eq 4 simplifies to:

$$\begin{array}{c} r_{ij}=\frac{\gamma }{M}g_{ij}\left(\theta_{j}\right)\frac{h_{j}\left(c_{j}\right)}{\sum_{\ell }h_{\ell }\left(c_{\ell }\right)}. \end{array}$$ (5)

Spiking activity was modeled as a homogeneous Poisson process, such that the probability of a neuron generating *n* spikes in time *T* was:

$$\begin{array}{c} \text{Pr}\left[n_{ij}\right]=\frac{{\left(r_{ij}T\right)}^{n_{ij}}}{n_{ij}!}\text{exp}\left(-r_{ij}T\right). \end{array}$$ (6)

Recall of the orientation of a probed item *p* was modelled as *maximum a posteriori* (MAP) decoding of feature information from the population’s spiking activity, **n**, over a decoding period *T*_*d*_. Assuming a uniform prior, this is equivalent to maximizing the likelihood:

$$\begin{array}{c} {\hat{\theta }}_{MAP}=\underset{\theta_{p}}{\arg\max}\mathrm{Pr}[n|\theta_{p}]. \end{array}$$ (7)

If two or more orientations tied for the maximum, the decoded value was sampled at random from the tied values. The output of the model was given by $\hat{\theta }=\theta_{MAP}⊕\beta$, where *β* is a response bias term, and ⊕ indicates addition on the circle. For simplicity we fixed the decoding period *T*_*d*_ to 1 s (changing this value would merely result in a corresponding change to the estimated gain parameter *γ*, e.g. setting *T*_*d*_ = 0.1 s would multiply the gain by 10).

In Exp 2, each stimulus consisted of a set of Gabor patches with varying orientation, and participants were required to store in memory the mean orientation of the patches. We assumed that the concentration (inverse variability) of the estimated mean was directly proportional to the concentration of the distribution from which the orientations were sampled, i.e.

$$\begin{array}{c} \theta_{j}∼\text{VM}\left(\mu_{j}^{\prime },s\kappa_{j}^{\prime }\right), \end{array}$$ (8)

where $\mu_{j}^{\prime }$ and $\kappa_{j}^{\prime }$ are the mean and concentration of the sampled distribution, *s* is a constant of proportionality, and VM(*μ*, *κ*) is a von Mises distribution with mean *μ* and concentration *κ*.

In order to fit the model to data, instead of relying on Monte Carlo simulation, we used a number of previously-obtained analytical results that provided a direct way of estimating the distribution of errors predicted under the model, on the assumption *M* → ∞; details of these methods can be found in Bays (2016); code is available at www.bayslab.com/code/JN14. In fitting the data from Exp 1, the model had five free parameters: *κ*, *γ*, *α*, *σ*, and *β*. In Exp 2, stimulus contrasts were fixed and identical for all stimuli, making parameters *σ* and *α* unnecessary, but the input orientations *θ*_*j*_ were now random variables controlled by the proportionality parameter *s*; the model therefore had four free parameters: *κ*, *γ*, *β* and *s*.

## Supporting information

S1 Text Supporting information. Comparison with models incorporating probabilistic encoding.
(PDF)

S1 Fig Model comparison. AIC scores relative to the score of the best fitting neural resource model. AIC values for simultaneous and sequential version of experiment are shown in blue and red, respectively.
(TIF)

S1 Table Maximum likelihood parameter estimates for simultaneous and sequential versions of Experiment 1. PE, probabilistic encoding model; PE-V, probabilistic encoding model with varying precision: PC-EF, population coding model with encoding front-end; PC-CT, population coding model with contrast threshold.
(TIF)
