# Supplementary material for: Internal but not external noise frees working memory resources
Source: PLoS Comput Biol. 2018 Oct 15;14(10):e1006488. doi: 10.1371/journal.pcbi.1006488 (PMC6201966; doi:10.1371/journal.pcbi.1006488)
Supplement: S1 Text — Comparison with models incorporating probabilistic encoding. (PDF) [file pcbi.1006488.s001.pdf]

Supporting Information

The results of Experiment 1 demonstrated that a manipulation that increased the influence of internal noise (reducing contrast) for one stimulus freed working memory to store another stimulus more precisely. We further showed that an existing model of working memory based on population coding [1, 2] could account for these behavioral results in quantitative detail. In the neural model, activity associated with the *variable noise* stimulus scaled with contrast following a Naka-Rushton function. This function was chosen in earlier work [2] because it provides a good approximation to the contrast responses of visual cortical neurons [3], as well as those earlier in the visual pathways, with few free parameters. Here we consider some alternative accounts of the relationship between stimulus contrast and signal strength, focusing in particular on models in which the *variable noise* stimulus is sometimes not encoded at all. Despite differences, all the following models adhere to the fundamental resource principle that attenuating (or eliminating) the signal associated with one stimulus results in an increase in signal strength for other stimuli, and they are therefore all consistent with the main conclusions of the study. In the event, none of these alternative models were supported over the model presented in the main text.

Probabilistic encoding model

The first model is the simplest in the class of "all-or-nothing" models (e.g. [4]) that might provide an alternative account for our data. Responses were derived from a probabilistic mixture of two types of trial, those on which the *variable noise* stimulus was encoded and those on which it was not. Assuming the relative frequency of these two kinds of trial varied with contrast, and if precision for the *low noise* stimulus was higher when the *variable noise* stimulus was not stored (according to the resource principle), this probabilistic model might produce the appearance of a continuous change in recall precision with contrast as observed empirically.

We assumed that *low noise* stimuli were always encoded, as were the matching (400% contrast) *variable noise* stimuli; stimuli with 0% contrast were by definition always unencoded. Stimuli of intermediate contrasts were encoded with varying probability, determined by free parameters $p_{75\%}$, $p_{100\%}$, $p_{150\%}$.

For encoded stimuli, we modelled recall responses as a circular normal (von Mises) distribution with precision that depended on the total number of encoded stimuli on that trial. For unencoded stimuli, responses were uniformly distributed. As a result, the distribution of responses for a *variable noise* stimulus with contrast C was a mixture with density:

$$p(\hat{\theta}) = p_C \mathrm{VM}(\hat{\theta}; \theta, \kappa_{both}) + \frac{(1 - p_C)}{2\pi}. \tag{1}$$

where $\mathrm{VM}(\theta; \mu, \kappa)$ is the density function of a Von Mises distribution with mean μ and concentration κ. The response distribution for a *low noise* stimulus was given by:

$$p(\hat{\theta}) = p_C \mathrm{VM}(\hat{\theta}; \theta, \kappa_{both}) + (1 - p_C)\mathrm{VM}(\hat{\theta}; \theta, \kappa_{single}). \tag{2}$$

We fit the model using the Nelder-Mead simplex method (*fminsearch* in MATLAB), and compared it to the population coding model using the Akaike Information Criterion. Maximum likelihood parameter estimates for this and subsequent models are shown in Table S1. The probabilistic encoding model was a poorer fit than the population coding model to data from both the simultaneous version of Exp 1 (ΔAIC = 188; 7 out of 9 participants; $t(8) = 3.13$, $p = 0.01$) and the follow-up sequential version (although with greater intersubject variability: ΔAIC = 14; 5 out of 10 participants; $t(9) = 0.349$). Combining results from simultaneous and sequential versions of the experiment by summing AIC scores, we obtained strong evidence in favor of the neural resource model (ΔAIC = 202).

Probabilistic encoding model with varying precision

We considered the further possibility that the precision of the *variable noise* stimulus, on those trials when it was encoded, varied with contrast. We supplemented the probabilistic encoding model with three free parameters, ($\{\kappa_{75\%}, \kappa_{100\%}, \kappa_{150\%}\}$, replacing κ_{both} in Eq. 1 above), that determined precision of the *variable noise* stimulus at intermediate contrasts (precision at the highest contrast was necessarily equal to κ_{both}, because the *variable noise* and *low noise* were physically indistinguishable in that condition; precision at 0% contrast was necessarily zero).

Compared to the neural resource model, the modified probabilistic encoding model was a poorer fit to the experimental data from both simultaneous (ΔAIC = 213; 8 out of 9 participants; t(8) = 3.76, p $<$ 0.01) and sequential (ΔAIC = 47; 6 out of 10 participants; t(9) = 1.2, p = 0.26) versions of Exp 1. In line with other comparisons, combining AIC scores provided strong evidence in favor of the neural resource model (ΔAIC = 260).

Population coding model with encoding front-end

We next turned our attention to models that attempted to incorporate the possibility of encoding failure into the neural model presented in the main paper. The first was a variant of the population coding model in which a probabilistic encoding process operated at the "front end". We again assumed that *low noise* stimuli and the matching (400% contrast) *variable noise* stimuli were always encoded. For intermediate contrasts, the probability of encoding the *variable noise* stimulus was determined by one of three free parameters $\{p_{75\%}, p_{100\%}, p_{150\%}\}$. To model an unencoded *variable noise* stimulus, we set the neural response (Eq. 1 in main text) of neurons dedicated to that stimulus to zero. Due to normalization, all the population activity was devoted to the *low noise* stimulus in these cases.

This model gave a consistently poorer account of the experimental data than the unmodified neural resource model for simultaneous (ΔAIC = 67; 9 out of 9 participants; t(8) = 3.98, p $<$ 0.01) and sequential versions of Exp 1 (ΔAIC = 57; 10 out of 10 participants; t(9) = 4.91, p $<$ 0.001). Combined evidence favored the unmodified neural resource model (ΔAIC = 124).

Population coding model with contrast threshold

Finally, we directly incorporated a threshold contrast for encoding into the neural model. We replaced the continuous contrast response function (Eq. 3 in main text) with a threshold function which output 1 if the contrast was above threshold (a free parameter) and 0 if below. This meant that the neural signal was either evenly distributed between stimuli or allocated solely to the *low noise* stimulus, depending on contrast.

The threshold model provided an inferior fit to the data compared to the unmodified neural

resource model, both in simultaneous (ΔAIC = 90; 9 out of 9 participants; $t(8) = 5.54$, $p < 0.001$) and sequential versions of Exp 1 (ΔAIC = 59; 6 out of 10 participants; $t(9) = 1.51$, $p = 0.17$). Combined AIC scores (ΔAIC = 149) again favored the unmodified neural resource model.

References

[1] Bays PM. Noise in Neural Populations Accounts for Errors in Working Memory. *Journal of Neuroscience* 34.10 (2014), 3632–3645.

[2] Bays PM. A signature of neural coding at human perceptual limits. *Journal of Vision* 16.11 (2016), 4.

[3] Albrecht DG and Hamilton DB. Striate cortex of monkey and cat: contrast response function. *Journal of Neurophysiology* 48.1 (1982), 217–237.

[4] Zhang W and Luck SJ. Discrete fixed-resolution representations in visual working memory. *Nature* 453.7192 (2008), 233–235.
