# Supplementary figures and images for: Internal but not external noise frees working memory resources
Source: PLoS Comput Biol. 2018 Oct 15;14(10):e1006488. doi: 10.1371/journal.pcbi.1006488 (PMC6201966; doi:10.1371/journal.pcbi.1006488)

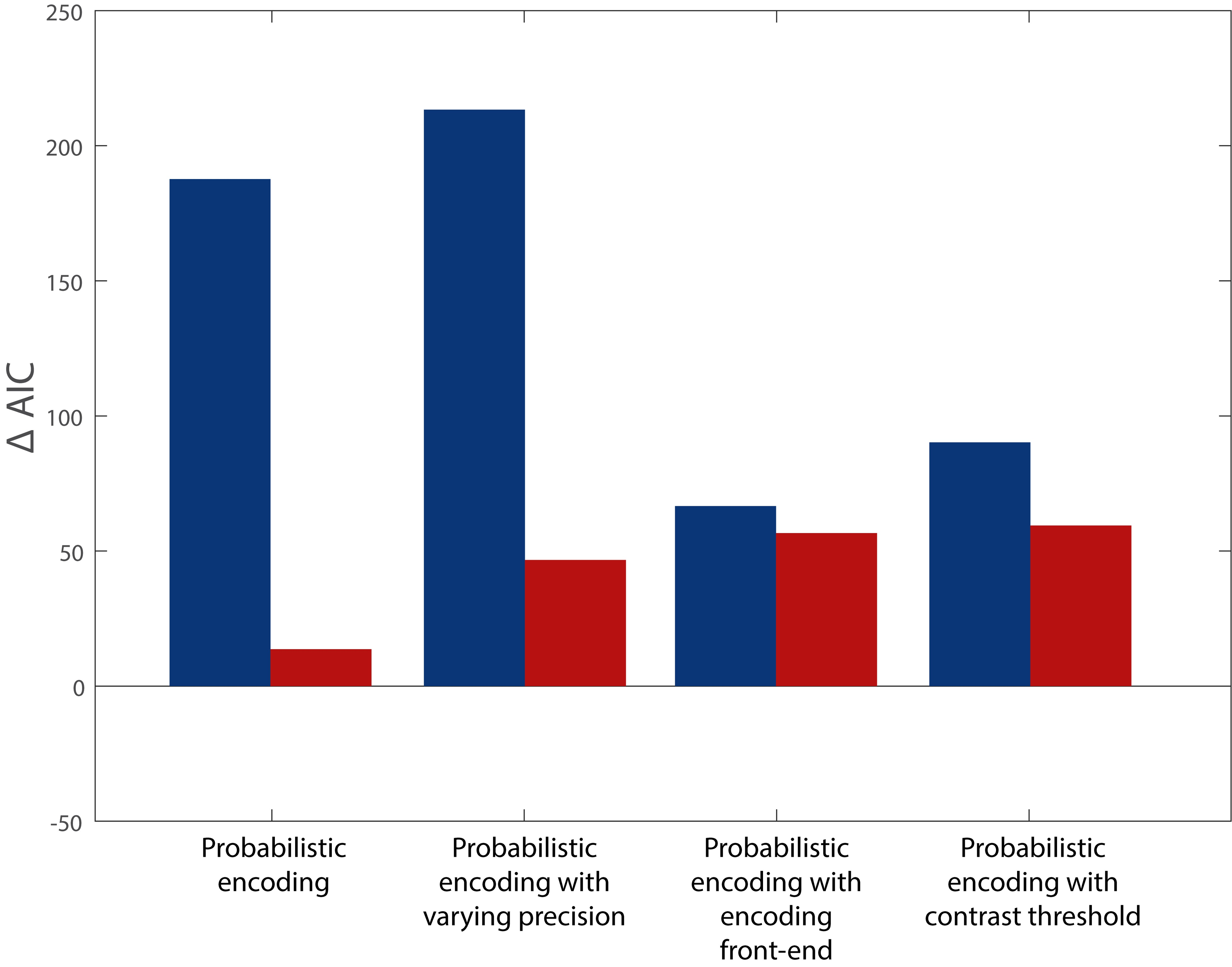

Supplement: S1 Fig — AIC scores relative to the score of the best fitting neural resource model. AIC values for simultaneous and sequential version of experiment are shown in blue and red, respectively. (TIF) [file pcbi.1006488.s002.tif]

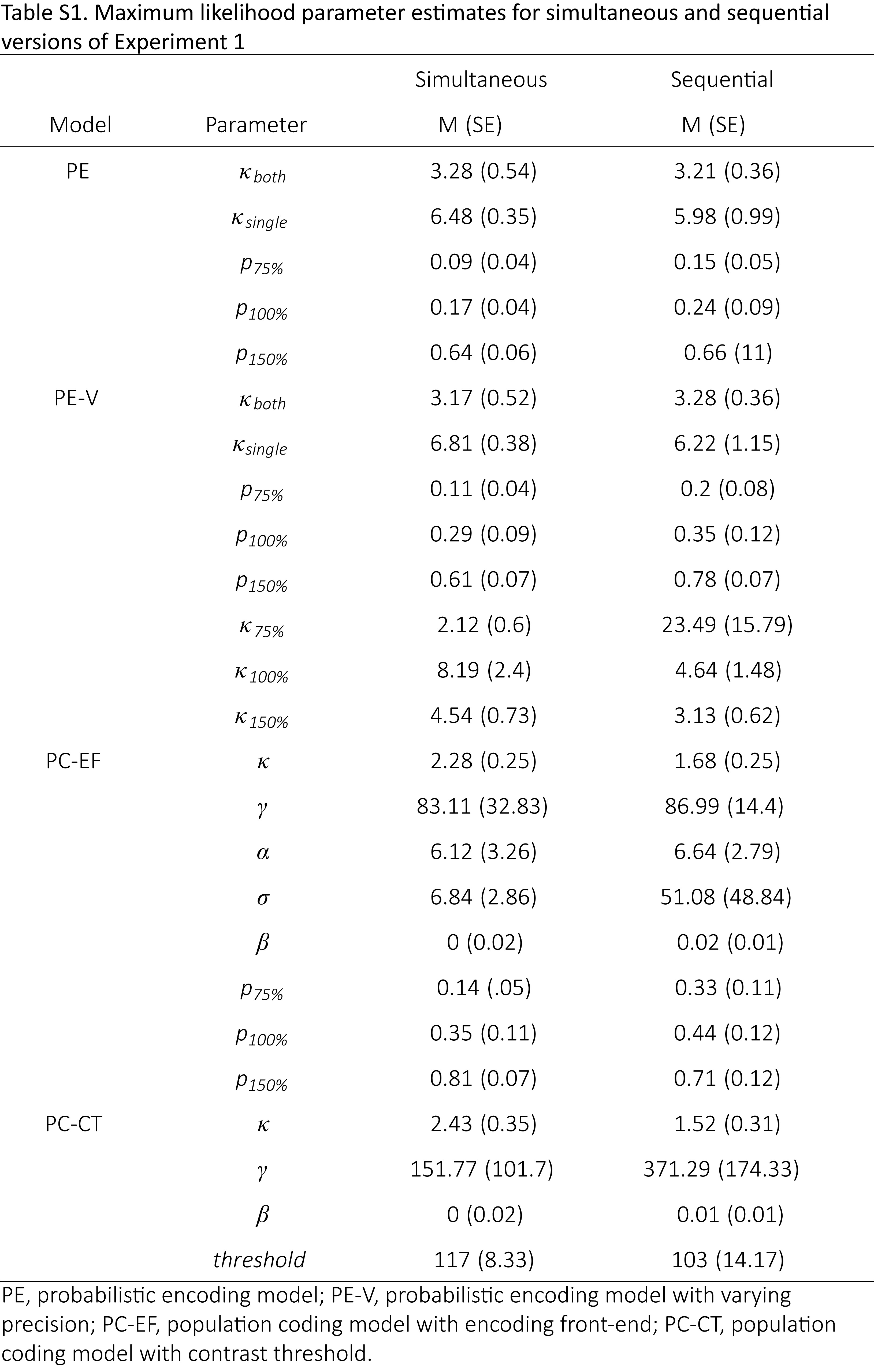

Supplement: S1 Table — PE, probabilistic encoding model; PE-V, probabilistic encoding model with varying precision: PC-EF, population coding model with encoding front-end; PC-CT, population coding model with contrast threshold. (TIF) [file pcbi.1006488.s003.tif]
